# Olive Pomace Extract Contains Low Molecular Weight Peptides and Possesses ACE Inhibitory Activity

**DOI:** 10.3390/ijms25073962

**Published:** 2024-04-02

**Authors:** Eduardo López-Huertas, Jose Rubí-Villegas, Lourdes Sánchez-Moreno, Rosa Nieto

**Affiliations:** 1 Group of Antioxidants and Free Radicals in Biotechnology, Food and Agriculture, Estación Experimental Zaidín (EEZ), Spanish National Research Council (CSIC), Profesor Albareda 1, 18008 Granada, Spain; 2 Instrumental Technical Services of the Estación Experimental Zaidín (EEZ), Spanish National Research Council (CSIC), Profesor Albareda 1, 18008 Granada, Spain; 3 Department of Nutrition and Sustainable Animal Production, Estación Experimental Zaidín (EEZ), Spanish National Research Council (CSIC), San Miguel 101, 18100 Armilla, Granada, Spain

**Keywords:** olive pomace, food peptides, bioactive peptides, angiotensin converting enzyme (ACE), peptidomics

## Abstract

The aim of the present study was to determine the ACE inhibitory activity of aqueous extracts of olive pomace and to understand whether they represent a good source of bioactive LMW peptides for nutritional and pharmacological applications. We produced a water extract from olive pomace (var. Picual) and obtained its low molecular weight (LMW) fraction (<3 kDa). The calculated yield of extraction was 100.2 ± 7.9 mg of LMW peptides per 100 g of olive pomace. The olive pomace LMW fraction possessed strong ACE inhibitory activity (IC_50_ = 3.57 ± 0.22 µg prot/mL). The LMW fraction (<3 kDa) was analysed by nanoscale liquid chromatography-Orbitrap coupled with tandem mass spectrometry and de novo sequencing. Thirty new peptides, containing between 7–17 amino acids and molecular masses ranging 778–1354 Da, were identified by the Peaks database algorithm using the available *Olea europaea* (cv. Farga) genome database. Ten new peptides were also identified by Peaks de novo sequencing. The protein sources of twelve peptides detected in the database by Peaks DB were identified by BLAST search. The ACE inhibitory activity of the identified peptides was predicted by BIOPEP software. We conclude that olive pomace possesses ACE inhibitory activity and contains low molecular weight peptides with (predicted) biological activity. Olive pomace may represent a good source of peptides for nutritional and pharmaceutical applications. In our study, it has been shown that olive pomace possesses ACE inhibitory activity and contains low molecular weight peptides with (predicted) biological activity. Olive pomace may represent a good source of peptides for nutritional and pharmaceutical applications. More research is needed in order to identify the in vivo effects of olive pomace bioactive peptides.

## 1. Introduction

Olive oil production is one of the main agrifood industries in Mediterranean countries such as Spain, Italy, and Greece [1]. Olive oil constitutes approximately 25% of the olive fruit, leaving the remaining 75% as disposable residue. Olive pomace is one of the main waste products of the olive oil industry. Olive pomace is a semi-solid viscous dark brown–violet coloured paste which contains water from vegetation, pulp, skin, crushed olive pits, and some residual oil [2]. The worldwide production of olive pomace is very high; it has been estimated at approximately 3 million tonnes per year [3]. The composition of olive pomace is characterised by high humidity (56–75%) and high fibre content (lignin, hemicellulose and cellulose), and also contains proteins, fat, water-soluble carbohydrates, polyphenols, pigments, and some minerals [2,4]. Olive pomace waste is harmful to the environment, as it negatively affects soil microbial flora, aquatic ecosystems, and the air because of its polyphenol content and sulphur dioxide emissions [5,6]. In addition, olive pomace is hardly biodegradable because of its high content of water and organic compounds [7].

To contribute to environmental sustainability, a number of strategies for the revalorization of olive oil waste have been proposed based on the extraction of compounds with added value that can be used for the food and pharmaceutical industries [8,9]. Such is the case for the recovery of antioxidant polyphenols [10], terpenic acids [11], polar lipids [12], protein hydrolysates [13,14], pigments (chlorophylls and carotenoids), tocopherols, phytosterols, squalene, and aromatic compounds [8]. Examples include the use of phenolic extracts of olive pomace to enrich vegetable oils, fish burgers, fermented milk, and in the coating of edible fruits to take advantage of its antioxidant and antimicrobial effects. It has also been used as a source of nutrients for the formulation of pasta, as a source of dietary fibre to enrich food, in animal nutrition, in the packaging industry, and in other applications [15]. The nutraceutical and pharmacological applications of olive pomace represent an active area of research [16,17].

High blood pressure is a primary risk factor of cardiovascular disease that affects approximately 40% of adults [18]. The angiotensin-converting enzyme (ACE) plays a key role in regulating blood pressure. Inhibition of ACE is a widely used strategy for the treatment of hypertension [19]. Indeed, ACE inhibitor drugs such as captopril and enalapril have an enhanced effect at lowering blood pressure and controlling heart failure. New sources of ACE inhibitors with antihypertensive activity are of interest for the food and pharmaceutical industries [20].

A high number of low molecular weight (LMW) peptides derived from foods have shown ACE-inhibitory and antihypertensive activities [21]. They are mostly produced via enzyme hydrolysis of proteins from animal or vegetable origins, although they can also occur naturally [22,23]. LMW peptides can be present in olive pomace as a consequence of the metabolism of olive fruit, or can originate as a result of the olive oil production process (olive milling, malaxation, centrifugation) by the action of the proteases and peptidases present in olive cells [24]. The use of olive pomace as a source of endogenous bioactive peptides for the food and pharmaceutical industries is a possibility that has not been investigated previously. Bearing in mind the large number of peptides with numerous functionalities described in foods, we hypothesised that olive pomace could contain endogenous peptides and possess angiotensin-converting enzyme (ACE) inhibitory activity.

In previous work carried out in our laboratory we have shown that virgin olive oil contains low molecular weight peptides with ACE inhibitory and antihypertensive activities [25]. The peptide sequences VCGEAFGKA, NALLCSNS, CPANGFY, CCYSVY, and DCHYFL [26] showed strong ACE inhibitory activities, while the peptides RDGGYCC and CCGNAVPQ demonstrated remarkable antihypertensive activity in spontaneously hypertensive rats [27]. However, olive oil contains low protein amounts and is not a good source for the extraction of antihypertensive peptides. The ACE inhibitory activity of olive pomace has not been investigated so far. The aim of the present study was to determine the ACE inhibitory activity of aqueous extracts of olive pomace and to understand whether they represent a good source of bioactive LMW peptides for nutritional and pharmacological applications. We report that olive pomace water extract possesses significant ACE inhibitory activity and may represent a good source of bioactive LMW peptides for nutritional and pharmacological applications.

## 2. Results and Discussion

### 2.1. Preparation of a Water-Soluble LMW Fraction from Olive Pomace Extract

The protein fraction of olive pomace has been poorly investigated to date. We obtained olive pomace from mature olives and optimised a water extraction method to produce an extract containing the LMW fraction (<3 kDa). The composition of this LMW fraction was then investigated. The crude protein content measured in fresh olive pomace was 2.54 ± 0.25 g per 100 g (determined as total nitrogen by the Dumas method; see Section 3.12). The protein content in clarified olive pomace extract (sn-3, resulting from the 100,000× *g* centrifugation step) was 0.53 ± 0.05 g per 100 g. The amount of protein measured in the LMW filtrate (<3 kDa) was 0.16 ± 0.02 g per 100 g of filtrate. The calculated yield of extraction was 100.2 ± 7.9 mg of LMW peptides per 100 g of olive pomace (range 90–110 mg/100 g), which represented approximately 4% of the protein content initially present in olive pomace. Our previous results showed that unfiltered virgin olive oil contained only between 0.3 and 1.2 mg of proteins per 100 g of oil [25], i.e., one hundred times fewer peptides than in olive pomace. Therefore, olive pomace was found to be a much better source of olive peptides compared with olive oil.

### 2.2. Amino Acid Analysis and Native PAGE of Olive Pomace LMW Extract

**Figure 1:**
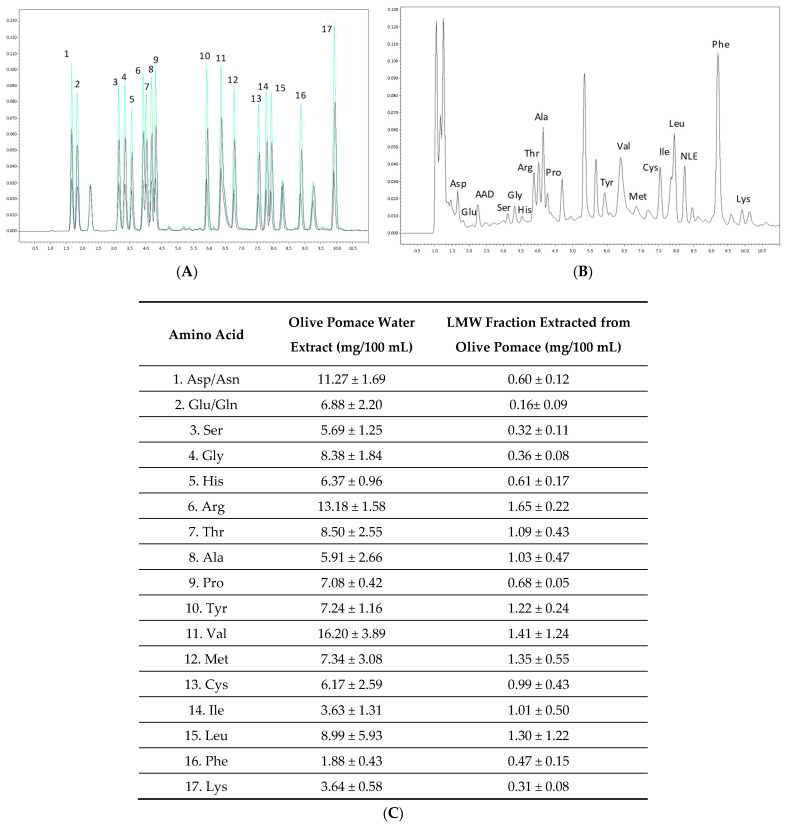
Amino acid profile measured by HPLC of olive pomace extract and its low molecular weight (LMW, <3 kDa) fraction: (**A**) mix of seventeen amino acid standards (at 0.1, 0.2, and 0.3 mM concentrations); (**B**) amino acid profile of the LMW fraction; (**C**) amino acid composition of olive pomace extract and the LMW fraction. AAD, L-alpha-amino adipic acid; NLE, DL-norleucine. The other large chromatographic peaks appearing in (**B**) originate from the phenyl isothiocyanate derivatising agent used in the analysis. Data are expressed as means ± standard deviation (SD).

Amino acids were analysed in olive pomace water extract (sn-3) and in the LMW fraction obtained from it. Figure 1 shows the amino acid profile obtained by HPLC (panel A, standards; panel B, LMW fraction) and the amino acid compositions of the olive pomace extract and the LMW fraction (Panel C).

Two different hydrolysis methods were used to determine the amounts of Met and Cys in the samples. The amounts of Glu and Asp shown in the table also contain Gln and Asn, as during acid hydrolysis Gln and Asn deamidate to produce Glu and Asp, respectively.

The difference between the protein amounts calculated by the Dumas method (total nitrogen content × 6.25) and the amounts calculated by the addition of individual amino acids indicates the content of non-protein nitrogen compounds. From the quantification of both amino acids and total nitrogen, the protein nitrogen fraction estimated in the LMW fraction obtained from olive pomace was 48.1% and the non-protein nitrogen was 51.9%, which is in agreement with previously reported values in industrial olive pomace waste [28]. Although the protein levels of the olive pomace extract and its LMW fraction were relatively low, their contents of essential amino acids were 49% and 59% of the total amino acids, respectively. Considering the extraordinarily high amounts of pomace olive generated in olive oil-producing countries [29], pomace water extracts could represent a possible source of essential amino acids.

**Figure 2:**
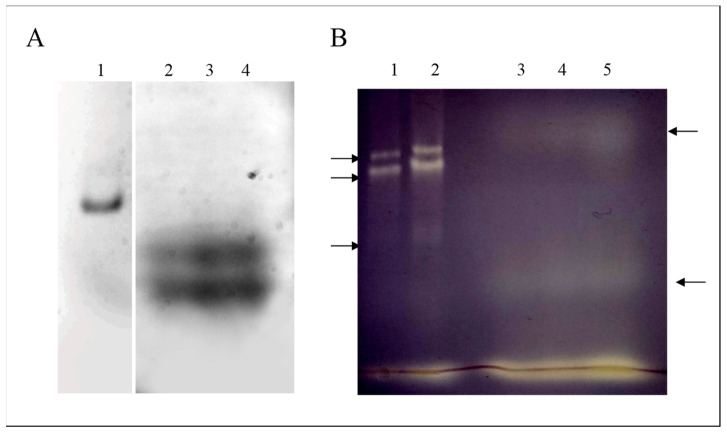
Native polyacrylamide gel electrophoresis of low molecular weight (LMW) peptide fraction extracted from olive pomace. Panel (**A**), protein staining with trihalo compounds. Lane 1, protein standard of bovine serum albumin (5 µg); lanes 2–4, olive pomace LMW fraction containing 2.5, 5, and 7.5 µg of proteins, respectively. Panel (**B**), superoxide dismutase (SOD) activity detected in LMW peptide fraction by a photochemical method using nitrobluetetrazolium. Lanes 1 and 2, SOD activity detected in an extract of *Olea europaea* showing (from top to bottom) isoenzymes of Mn-, Fe- and Cu,Zn-SOD (control); lanes 3, 4, and 5, olive pomace LMW fraction containing 2.5, 5, and 7.5 µg of proteins, respectively, showing bands of superoxide dismutase antioxidant activity (see arrows).

The peptide content of olive pomace LMW fraction was visualised by native PAGE using trihalo polyacrylamide gels (Figure 2). Two large undefined bands of (tryptophan-containing) peptides were detected in the gels, which was somewhat expected, as all of the peptides were similar in size (<3 kDa). In native PAGE, small peptides migrate mostly in accordance with their size and their native charge, which is usually negative in alkaline buffer (pH 8.3). The antioxidant activity of the LMW fraction was also investigated using a specific superoxide dismutase staining method developed in native gels. Several superoxide anion radical quenching bands (areas) were observed in the gels, suggesting antioxidant activity of peptides occurring in the LMW fraction. This result is supported by our previous research describing peptide sequences with antioxidant activity in olive oil [26]. Finally, an intense superoxide dismutase band was observed with the dye at the bottom of the gel, most likely due to the antioxidant activity of polyphenols (see later).

### 2.3. Fractionation of Peptides by Size-Exclusion Chromatography

**Figure 3:**
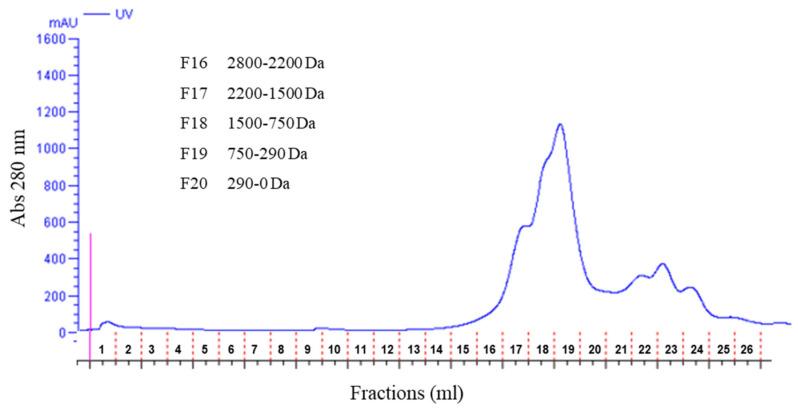
Fractionation of LMW peptides extracted from olive pomace by gel filtration chromatography and FPLC. F, fraction.

The fractionation of the LMW extract obtained from olive pomace by gel filtration chromatography (FPLC) is shown in Figure 3. Peptides and polyphenols both absorbed at 280 nm; therefore, the UV absorption profile was not useful for identifying peptide fractions. However, the molecular mass calibration of the column indicated that fractions 17–18 (2200–750 Da) contained the majority of the peptides, while fractions 19–20 (750–0 Da) included the bulk of polyphenols (usually <700 Da) as well as peptides and other molecules. Fraction 20 (<290 Da) contained small phenolic compounds, dipeptides, tripeptides, free amino acids, and other small molecules. The total content of polyphenols measured in the LMW extract was 8.2 mg/mL, which is in agreement with previously reported values detected in olive pomace (ranging from 1–23 mg/g of fresh olive pomace) [30,31,32]. Polyphenols were undetectable in fractions 16 and 17, but were mostly present in fractions 19 and 20. 

### 2.4. ACE Inhibitory Activity Determinations

**Figure 4:**
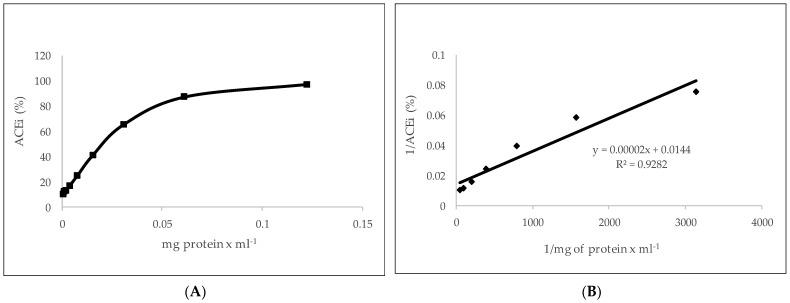
(**A**) Angiotensin-converting enzyme inhibitory activity (ACEi) of water-soluble LMW peptides extracted from olive pomace and (**B**) calibration curve obtained from the data of panel A and used to determine IC_50_. Data are expressed as the mean of three experiments.

The ACE inhibitory activity was investigated in pomace water extract of low molecular weight, as antihypertensive peptides typically possess molecular sizes <3 kDa [33]. The ACE inhibitory activity of the LMW olive pomace extract and FPLC-purified fractions (16–20) is shown in Table 1 and Figure 4. The LMW extract showed strong ACE inhibitory activity (IC_50_ 3.57 ± 0.22 µg prot/mL of pomace extract), similar to that previously reported by our group in olive oil extract [25].

We investigated the possible contribution of olive polyphenols to the overall ACE inhibitory activity. Certain olive polyphenols, such as oleuropein aglycone, ligstroside aglycone, and oleoside extracted from olive leaves, have been previously reported to possess ACE (partial) inhibitory activity at millimolar concentrations [34]. Interestingly, the ACE inhibitory activity of the polyphenol extract obtained from the LMW fraction (see Section 3.5) did not show any activity. ACE inhibitory activity was detected in the FPLC fractions 16–20, particularly in fractions 18–20 (Table 1). The activity detected in fractions 16–18 was most likely due to the ACE inhibitory peptides present in the olive pomace LMW extract (see later). Fractions 19 and 20 (<750 Da) contained most of the polyphenols, and their contribution to the ACE inhibitory activity cannot be ruled out. A good number of dipeptide and tripeptide species (<500 Da) usually possess ACE inhibitory activity [35], and may also have contributed to the overall inhibitory activity.

### 2.5. Endogenous Peptides and Proteins Identified by Peaks DB and De Novo Sequencing in Olive Pomace

Mass spectrometry and PEAKS Studio 10.6 software [36,37] were used to identify the peptides present in olive pomace LMW extract. Using the available *Olea europae* database, Peaks DB algorithm identified 30 endogenous peptides with −10lgP score > 25 that had not been reported before (Supplementary Materials Table S1). A selection of twelve peptide sequences containing accession numbers for the proteins containing the peptides (detected by BLAST search) is shown in Table 2. The identified peptides possessed between seven and twelve amino acids and molecular masses ranging from 778 to 1141 Da. 

The presence of hydrophobic or basic charged amino acids such as arginine or lysine was detected within the last three amino acids of the C-terminal position of most of the peptides. ACE-inhibitory peptides usually contain hydrophobic and/or basic charged amino acids at the C-terminal position [37]. In addition, more than 50% of the identified peptides contained cysteine residues, which are usually involved in redox reactions with antioxidant potential [38]. The antioxidant activity of peptides has been attributed to certain sequences of amino acids containing cysteine, methionine, lysine, histidine, tryptophan, and tyrosine [39,40]. The presence of hydrophobic amino acids has also been described in peptides with antioxidant activity [41], and may present advantages because of their easier access to hydrophobic molecules such as fatty acids [42].

The proteome of *Olea europaea* is mostly uncharacterised (only 112 reviewed sequences in Swiss-Prot), and the available genomic and transcriptomic databases mostly contain computationally analysed records. Thirteen proteins were reported as sources of the peptide sequences identified by Peaks DB (Supplementary Materials Table S2). The *Olea europaea* database used in these analyses was unannotated; thus, although the accession numbers for the proteins containing the peptides were obtained by Peaks DB, the protein identification was not provided. BLAST searches against the nonredundant protein sequences database at NCBI were carried out in order to identify the protein sources of the peptides detected in olive pomace.

Ten novel peptides (not present in the olive database) with ALC% above 86 were confidently identified by the Peaks de novo sequencing algorithm (Table 3). The detected peptides contained between six and twelve amino acids and were mostly rich in hydrophobic residues (>50%). Five peptides contained histidine and/or cysteine in their sequences. The presence of antioxidant peptides in olive fruit and olive oil may have implications for its oxidative stability.

### 2.6. Predicted Activities of Olive Pomace Endogenous Peptides

The biological activity of the peptides identified in olive pomace by Peaks DB and the Peaks de novo sequencing algorithm was investigated using the BIOPEP database of bioactive peptides (Table 4 and Table 5, respectively). According to BIOPEP, 38 peptides possessed ACE-inhibitory activity, while the peptides MCTAAEMK and (de novo) YCFHSAA had previously described hypotensive potential [43]. As mentioned before, ACE inhibition is a target for the treatment of hypertension. All peptides showed dipeptidyl peptidase (DPP)-III- and/or IV-inhibitory activity. DPP-III plays a role in blood pressure regulation by modulating angiotensin I and II [44]. Human DPP-IV is involved in blood glucose regulation, and its inhibition is used as a target for antidiabetic drugs [45]. Other biological activities detected in the peptides were antithrombotic, anti-inflammatory, renin inhibitor, glucose uptake stimulator, regulator of stomach mucosal activity, anti-amnestic, alpha-glucosidase inhibitor, Calmodulin-dependent cyclic nucleotide phosphodiesterase (CaMPDE) inhibitor, activator of ubiquitin-mediated proteolysis, and regulator of phosphoglycerate kinase activity. These predicted biological activities indicate potential nutritional and pharmaceutical applications, and could be the subject of further studies.

As mentioned before, we previously showed that virgin olive oil contains LMW peptides with ACE-inhibitory and antihypertensive activities [25,26,27], although at a low concentration; in contrast, olive pomace constitutes an alternative and richer source of LMW peptides with ACE-inhibitory activity, and perhaps with other bioactivities yet to be explored. The ACE inhibitory activity of olive pomace could be a possibility for creating added value from olive waste and diversifying its applications, thereby improving its use and profitability while reducing the environmental problems related with this residue. This could be relevant when implementing circular economy strategies to improve the environment. The antihypertensive effects of olive pomace LMW peptides are currently being tested in vivo using a spontaneously hypertensive rat model of hypertension.

## 3. Materials and Methods

### 3.1. Plant Material and Extraction of Olive Oil

Olive fruits (*Olea europaea* L. variety Picual) were obtained from heathy olive trees at the Experimental Orchard of the Instituto de Investigación y Formación Agraria y Pesquera (IFAPA, https://www.juntadeandalucia.es/agriculturaypesca/ifapa/web/, URL accessed on 14 March 2024), “Centro Venta de Llano” located in Mengibar (province of Jaén), Spain. The olive samples were hand-picked when the olives were at the mature phase of ripening (ripening index 5 and 6) according to the scale described in [46], defining the degree of ripening depending on the colour of the skin and pulp of the olive. To obtain olive pomace, the selected olives were first washed thoroughly with water and subsequently dried with filter paper. A two-phase semi-industrial continuous system for olive oil extraction (Molinetto^®^, Pieralisi Maip SpA, Jesi, Ancona, Italy) which separates olive oil from wet olive pomace (named “alperujo”) was used. The use of water during the olive oil/olive pomace extraction was always avoided. The olive pomace was collected halfway through the extraction process in sealable bags. The samples were then frozen at −80 °C until further use to prevent degradation of bioactive compounds.

### 3.2. Preparation of Olive Pomace Extract Containing Low-Molecular Weight Peptides

Olive pomace peptides were extracted from our olive pomace preparations in several batches with only Milli-Q water using an olive pomace-to-water ratio of 1:2 (*w*/*v*). The mixture was homogenised in a blender for 30 s and then filtered through three layers of miracloth (previously rinsed with distilled water and Milli-Q water) to obtain the olive pomace crude water extract, which was centrifuged in 50 mL Falcon tubes at 1000× *g* for 10 min at 4 °C. The supernatant (sn-1) contained a (solid) olive oil layer on the top, which was carefully set aside to allow the liquid supernatant to be collected onto new fresh tubes; this was then centrifuged at 20,000× *g* for 20 min at 4 °C in a Sorvall RC 5C Plus centrifuge. The supernatants (sn-2) were then transferred to polycarbonate tubes and spun at 100,000× *g* at 4 °C in a Beckman ultracentrifuge using a 60Ti fixed angle rotor. The resulting clarified supernatants (sn-3) were loaded onto Vivaspin^®^ 6 centrifugal concentrators (ref. Z629456, Sigma-Aldrich Inc., St. Louis, MO, USA) with a 3 kDa cutoff (polyethersulfone) membrane. The tubes were centrifuged at 3500× *g* in a swing bucket rotor for 24 h. The filtrates obtained from this step contained the water extracted compounds with a molecular mass below 3 kDa, including the low molecular weight (LMW) peptide fraction. The filtrates were collected and stored at −80 °C until needed.

### 3.3. Analysis and Quantification of Amino Acids

The amino acid content of olive pomace and the LMW peptides (<3 kDa) extracted from olive pomace was determined after hydrolysis in 6 N HCl plus 1% phenol in sealed Pyrex tubes (Corning, NY, USA) at 110 °C for 24 h by high-performance liquid chromatograph (HPLC) according to the Waters Pico Tag method [47] as described in [48], using a Waters 2695 separation module (Waters Corporation, Mildford, MA, USA). L-alpha-amino adipic acid and DL-norleucine were used as internal standards. Pre-column derivatization was carried out with phenyl isothiocyanate. The cysteine and methionine contents were determined as cysteic acid and methionine sulphone, respectively, after oxidation of samples with performic acid prior to the protein hydrolysis step, as described in [49]. For quantification, seventeen amino acid standards (Amino Acid Standard H, Thermo Fisher Scientific, Waltham, MA, USA), L-Cysteic acid, and L-Methionine sulfone (Sigma-Aldrich, St. Louis, MO, USA) were used at concentrations of 0.1, 0.2, and 0.3 mM. Tryptophan was not determined. A Millennium 32 chromatography manager system was used for gradient control and data processing. Each sample was analysed three times with two technical replicates.

### 3.4. Detection of Peptides and Antioxidant Activity of Olive Pomace LMW Peptides in Native Polyacrylamide Gels

Olive pomace LMW samples were subjected to native-polyacrylamide gel electrophoresis (PAGE) in 4–20% gradient precast polyacrylamide gels (Mini-Protean TGX Stain-Free gels, Biorad^®^, Hercules, CA, USA) containing trihalo (2,2,2-Trichloroethanol) [50]. During electrophoresis, trihalo covalently modifies tryptophan residues in the protein, generating a fluorescence signal which can be observed with UV light. Peptide bands were visualised in a UV transilluminator. For the detection of superoxide dismutase (SOD) activity in gels, peptide samples were first separated by native-PAGE on 10% acrylamide gels. SOD activity was detected by a photochemical method using nitrobluetetrazolium [51]. A freshly prepared *Olea europaea* extract [24] containing the isoenzymes Mn-, Cu,Zn-, and Fe-SOD was used as a control.

### 3.5. Extraction of Polyphenols from Olive Pomace Extracts

First, 1 mL of the sample was mixed with 5 mL of ethyl acetate, vortexed for 30 s, and centrifuged at room temperature at 10,000× *g* for 10 min. The supernatant was then separated, the lower aqueous phase was extracted again with 5 mL of ethyl acetate following the same procedure, and both supernatants were combined. The resulting mix was evaporated under vacuum and the residue was reconstituted in methanol and filtered through a 0.20 μm PTFE syringe filter. The methanol extract was used for quantification purposes. All of the extractions were performed in duplicate, and the solutions were kept at −20 °C until analysis.

### 3.6. Analysis of Minerals by Inductively Coupled Plasma-Optical Emission Spectrometry (ICP-OES)

One gram of the freeze-dried LMW olive pomace extract samples was weighed accurately and freeze-dried. The resulting samples were mixed with 4 mL of 48.75 (*w*/*w*) of HNO_3_ and subjected to microwave digestion (Ultrawave MA149-015, Milestone, Sorisole, Italy) for one hour. The samples were then made up to 25 mL with milli-Q water and analysed in triplicate by ICP-OES using an Agilent 75800 ICP-OES optical emission inductively coupled plasma spectrometer with dual view (Agilent Technologies, Santa Clara, CA, USA). The instrument was calibrated with 26 elements (ICP multielement calibration standard solution, Scharlau, Spain) by setting the correlation coefficient limit at ≥0.999. Yttrium standard for ICP (Sigma-Aldrich INC, St. Louis, MO, USA) in 2% HNO_3_ was used as the internal standard. The instrument running conditions and wavelength of the spectrometer for each element were similar to those mentioned in [52].

### 3.7. Fractionation of Olive Pomace Peptides by Size-Exclusion Chromatography

The olive pomace LMW peptide (<3 kDa) fraction obtained with the method described above was separated by fast protein liquid chromatography (FPLC) using gel filtration chromatography with the protein purification system “ÄKTApurifier” (GE Healthcare, Chicago, IL, USA) equipped with a Superdex Peptide 10/300 GL size-exclusion column with a separation range of between 7000 and 100 Da (GE Healthcare, Chicago, IL, USA). The elution of the samples was conducted using an isocratic method with a mobile phase of 25 mM Tris-HCl pH 7.5 with 75 mM NaCl and a flow of 0.4 mL/min for 60 min. The elution was monitored at 280 nm. Standard proteins of known molecular mass were used to calibrate the column. The proteins used were cytochrome C (12,384 Da), aprotinin (6512 Da), Vitamin B12 (1355 Da), and the synthetic peptide YEAYEAS (725 Da). The injection volume of the samples and standards into the ÄKTApurifier was 100 µL. The protein content of the samples injected in the column, obtained from the amino acid quantification, was adjusted to 250 µg. Twenty-eight fractions of 1 mL (bed volume) were collected after each sample fractionation. The method was highly reproducible and produced identical sample chromatograms; thus, the same tubes were used to collect fractions from fifteen fractionation runs. The fractions were dried using an R-205 Buchi rotary evaporator (BÜCHI Labortechnik AG, Flawil, Switzerland) and resuspended in 25 mM Tris-HCl with a pH of 8. The fractions were analysed for ACE inhibitory activity, protein concentration, and polyphenol concentration.

### 3.8. Determination of Angiotensin-Converting Enzyme Inhibitory Activity

The ACE inhibitory activity was determined according to the method in [53] as described in [26]. This assay is based on the ability of ACE to hydrolyse the substrate o-aminobenzoylglycyl-p-nitrophenylalanyl-proline (Abz-Gly-Phe-(NO2)-Pro, Bachem Feinchemikalien, Switzerland), producing the fluorescent product o-aminobenzoylglycine (Abz-Gly). The following reagents were used: buffer A, 150 mM Tris-HCl buffer (pH 8.3) with 0.1 µM ZnCl_2_; buffer B, 150 mM Tris-HCl buffer (pH 8.4) with 1125 mM NaCl; ACE solution, rabbit lung ACE (E.C.3.4.15.1., Sigma-Aldrich) previously dissolved in 50% glycerol was diluted in buffer A to make up an enzyme concentration of 0.042 U/mL. This solution was prepared freshly every day to conduct the experiment. For the substrate solution, Abz-Gly-Phe(NO2)-Pro was dissolved in buffer B to a final concentration of 0.45 mM. This solution was also prepared every day before use, and was protected from light and kept at 4 °C. The assay was carried out using a fluorescence technique. Black polystyrene 96-well plates (Thermo Scientific, San Jose, CA, USA) were used. The wells contained the following reaction solutions: control = 40 μL of Milli Q water and 40 μL of ACE solution; blank = 40 μL of Milli Q water and 40 μL of buffer A; sample = 40 μL of LMW pomace extract and 40 μL of ACE solution; blank sample = 40 μL of sample and 40 μL of buffer A. The enzymatic reaction was initiated by adding 160 μL (final volume in each well 240 μL) of substrate solution and then immediately mixing the plate and incubating it at 37 °C in a VICTORX5 fluorimeter (PerkinElmer, Shelton, CT, USA). The fluorescence generated in this way was measured after 30 min using 355 nm and 420 nm as the excitation and emission wavelengths, respectively. The ACE inhibitory activity of each sample was determined in triplicate. The ACE inhibitory activity was calculated using the following formula.

$$ACE\;inhibitory\;activity\left(\%\right)=\frac{\left(FC-FB\right)-\left(FS-FBs\right)}{FC-FB}\times 100$$

In the formula, FC (control) is the fluorescence emitted after the action of ACE on the substrate without the inhibitor (i.e., sample); FB (blank) is the fluorescence emitted by the substrate; FS (sample) is the fluorescence emitted after the action of ACE on the substrate with the inhibitor sample; and FBs (blank sample) is the fluorescence emitted by the substrate and the sample. The ACE inhibitory activity was expressed as the IC_50_, which is the concentration of inhibitor required to inhibit the activity of ACE by 50%.

To determine the ACE inhibitory activity of the polyphenols from the LMW extract, extraction was carried out as described in Section 3.5. The resulting methanol extract was evaporated in a speed vacuum, then the residue was resuspended in 1 mL of buffer A prior to the test.

### 3.9. LC-Orbitrap MS/MS Analysis of Olive Pomace LMW Peptide Fraction

The peptide LMW fraction extracted from olive pomace described above was analysed by nanoscale liquid chromatography-Orbitrap coupled with tandem mass spectrometry (nanoLC-Orbitap-MS/MS) and de novo sequencing. For this, three samples of olive pomace water extract (<3 kDa) obtained as described above were freeze-dried and the powder was dissolved in 1% trifluoracetic acid in Milli-Q water. The samples were then centrifuged at 200,000× *g* for 1 h in Beckman a TL-100 ultracentrifuge using a TLA 100.2 rotor. Next, 5 μL of samples (containing 1 μg of proteins) were analysed by nanoLC-Orbitrap MS/MS in duplicates. The peptides were separated onto a ThermoEASY-Spray C18 PepMap^®^ column (75 μm × 50 cm, 2 μm particle size, 100 Å pore diameter) coupled to a trap nanocolumn (ThermoScientific Acclaim PepMap^®^100, C18) (3 μm, 100 Å, 75 μm × 2 cm). Chromatographic separation was performed as described in the EASY-nLC 1000 nano-LC system (Thermo Scientific, San Jose, CA, USA) using 0.1% formic acid in milli-Q water (phase A) and 0.1% formic acid in acetonitrile (phase B) with the following gradient: 0 to 8% B in 1 min, 8 to 21% of B in 130 min, and 21 to 90% B in 6 min. A flow rate of 200 nL/min was used to elute peptides for real-time ionisation and peptide fragmentation was performed using a Q-Exactive Orbitrap (Thermo Scientific) mass spectrometer. An enhanced Fourier transform MS/MS-resolution spectrum (resolution = 30,000 full width at half-maximum) was obtained, followed by a data-dependent MS/MS scan. The data-dependent MS/MS event consists of collision-induced dissociation fragmentation (35% normalized collision energy) and ion trap-MS/MS acquisition from the most intense ten parent ions with a charge state rejection of +1 (Z = 1) and dynamic exclusion of 0.5 min, which is typically used for peptide identification. Interference with other possible organic compounds was reduced by selecting only precursor ions with a minimum charge of +2 (Z = 2) or higher.

### 3.10. Peptide and Protein Identification by Database Search and De Novo Sequencing

PEAKS Studio 10.6 software [36,37] (Bioinformatics Solutions Inc., Waterloo, ON, Canada) software was used for the treatment of the raw mass spectrometry data obtained from the nanoLC-Orbitrap-MS/MS analysis. Peaks database searching-based protein identification (Peaks DB) and de novo sequencing were used to identify the most likely peptide sequence that matched the resulting spectra obtained from the LC-MS/MS analysis [36]. The following parameters were used for the analysis: parent mass error tolerance 10.0 ppm; fragment mass error tolerance 0.5 Da; precursor mass search type: monoisotopic; enzyme: none; variable modifications: oxidation of methionine. De novo peptide sequencing results obtained by Peaks were confirmed by local confidence scores at the amino acid level. The local confidence score ranges from 0% to 99%, showing how confident the algorithm considers a particular amino acid in the sequence as the correct assignment. The results were filtered using the average local confidence (ALC) parameter, which is the average of the local confidence score of all the amino acids in the sequence. An ALC value above 86% was used to filter the results obtained from de novo sequencing analysis. Database searches were carried out with the Peaks DB algorithm using the available *Olea europaea* (cv. Farga) genome database [54]. This database contains 55,595 protein-coding genes, and can be downloaded at https://denovo.cnag.cat/olive, URL (accessed on 1 December 2023). Peptide–spectrum matches were filtered by peptide −10lgP score. This parameter is calculated for every peptide–precursor spectrum match reported by Peaks DB, and indicates the statistical significance of the peptide–precursor spectrum match (i.e., the quality of the match). The score is derived from the *p*-value, defined by Peaks as the probability of a false identification in the database search achieving the same or better matching score. For example, a −10lgP score of 20, suggested by Peaks as a starting value to filter peptide–spectrum matches [55,56], is equivalent to a *p*-value of 0.01, which indicates that the probability of the peptide amino acid sequence being false is ≤ 1%. In our study we used a −10lgP score threshold of 25 (*p* = 0.003) to filter the peptide–spectrum matches. Identified proteins were filtered using a −10lgP score of 30. The protein score is calculated as the weighted sum of the −10lgP scores of the protein’s supporting peptides. The relative abundance of peptides was calculated by Peaks based on peptide features detected from LC-MS/MS data by integrating the area under the curve (label free quantification). Protein abundance was calculated by addition of the areas obtained from the supporting peptides. The protein sequences retrieved from the database search were subjected to NCBI BLAST search [57] to look for significant alignments and similarities with other previously identified proteins present in their *Olea europaea* database. Those protein identification matches of the highest quality (with identity percentages of 100% and cover percentages >95%) were reported.

### 3.11. Biological Activity Prediction of Identified Peptides from Olive Pomace Peptides

The biological activities of the peptides obtained by de novo sequencing and database search were explored by using the BIOPEP-UWM database of bioactive peptides [58]. This database contains referenced data from 4800 bioactive peptides (as of February 2024), including 1110 ACE inhibitors and 839 antioxidant peptides [59]. Potential biological activity of the peptide sequence is calculated by the database using parameters A and B. Parameter A is the number of sequences with the previously reported activity divided by the total number of amino acid residues (N) of the peptide sequence. Parameter B is the potential biological activity of the peptide (µM^−1^) calculated using published data of the activity of sequence motif(s) present in the peptide [60].

### 3.12. Determination of Total Nitrogen, Fat, Carbohydrates, and Polyphenols

The content of total nitrogen in olive pomace and LMW extract containing peptides (<3 kDa) was determined by the Dumas method ([61], AOAC method 990.03) in a Leco TruSpec CN analyser (LECO, St. Joseph, MI, USA). Crude protein content was calculated using a factor of ×6.25. Total polyphenols were measured as described in [62]. Fat content in olive pomace extracts was determined using Soxhlet extractor for 8 h with hexane using method 5520-D, as described elsewhere. Sugars were determined by the Luff–Schoorl method [63]. Starch was quantified as in [64]. Each sample was analysed three times with two technical replicates.

### 3.13. Statistical Analysis

Data are expressed as means ± standard deviation (SD). SPSS statistical software version 26.0 was used (SPSS, Chicago, IL, USA).

## 4. Conclusions

In this study, it has been shown that olive pomace possesses ACE inhibitory activity and contains low molecular weight peptides with (predicted) biological activity. Olive pomace may represent a good source of peptides for nutritional and pharmaceutical applications. More research is needed in order to identify the in vivo effects of olive pomace bioactive peptides.

## Figures and Tables

**Table 1:** ACE inhibitory activity (shown as IC_50_) of olive pomace water-soluble LMW fraction containing peptides and of FPLC-purified fractions (F) 16 to 20. Data are expressed as mean values ± SD.

Sample	IC_50_ (µg prot/mL)
Olive pomace LMW fraction	3.57 ± 0.22
F16	365.8 ± 32.3
F17	125.1 ± 33.1
F18	75.6 ± 6.9
F19	67.4 ± 14.5
F20	68.52 ± 7.49

**Table 2:** Endogenous peptides identified in olive pomace using Peaks database (DB) algorithm with accession numbers of the *Olea europaea* proteins from which they originate. −10lgP score, statistical significance of the peptide–precursor spectrum match; N, number of amino acids; *m*/*z*, precursor mass-to-charge ratio; RT, retention time; ppm, precursor mass error; Area, peptide abundance shown as area under the curve of the detected peptide; Protein source, best-matched protein identified by BLAST search (*Olea europaea*) and accession number of the protein; Accession, the accession number of the highest-scoring protein containing this peptide. Oxidised methionine is indicated by M(+15.99). ND, not detected.

No.	Peptide Sequence	−10lgP	N	*m*/*z*	RT	ppm	Area	Protein Source (BLAST)	Accession
1	NSNALACCSA	34.52	10	477.1920	51.20	7.1	3.16 × 10^7^	anthocyanidin 3-O-glucosyltransferase	OE9A062433P1
								CAA2981081.1	
2	GGDNGMNH	31.98	8	401.1449	51.36	−4.5	4.85 × 10^5^	phospholipase D gamma 1-like	OE9A085147P3 OE9A085147P2
								CAA2951337.1	
3	RSCQHRY	30.05	7	475.2191	21.09	0.4	ND	putative RING-H2 finger protein ATL21A	OE9A113191P1
								XP_022855964.1	
4	LPPDNSH	29.26	7	390.1841	52.42	2.7	3.26 × 10^6^	Hypothetical predicted protein	OE9A032896P1
								CAA2978878.1	
5	SLIPCCSS	27.21	8	809.3359	54.15	−6.6	6.69 × 10^6^	casein kinase 1 HD16	OE9A059381P2 OE9A059381P1 OE9A059381P3
								CAA2934848.1	
6	SGDPTM(+15.99)YE	26.79	8	458.1616	50.95	−11.8	2.68 × 10^5^	tryptophan aminotransferase-related 2 isoform X1	OE9A048079P1
								CAA2969551.1	
7	IDHGCAKH	25.50	8	440.7025	42.72	−1.2	1.65 × 10^6^	anthocyanidin 3-O-glucosyltransferase	OE9A062433P1
								CAA2981081.1	
8	IPATDSQG	25.47	8	394.6899	36.41	4.6	1.42 × 10^6^	auxin response factor 6 isoform X1	OE9A083314P2 OE9A083314P3 OE9A083314P1
								CAA2976018.1	
9	MCTAAEM(+15.99)K	25.21	8	450.6838	48.32	9.9	4.66 × 10^5^	white-brown complex homolog 30	OE9A053685P3
								CAA2969019.1	
10	GGGPGGGLGGPS	25.13	12	435.2055	45.44	3.6	3.10 × 10^6^	dihydrolipoyllysine-residue succinyl transferase component of 2-oxoglutarate de-hydrogenase	OE9A068558P1
								CAA3014561.1	
11	QANACHH	25.10	7	390.6582	33.30	−2.5	2.59 × 10^5^	DUF946 domain-containing DUF1162 domain-containing Chorein	OE9A008961P3
								CAA2957578.1	
12	NSCCVCMVR	25.00	9	507.6946	42.64	−4.6	1.31 × 10^7^	probable E3 ubiquitin-ligase LOG2	OE9A009309P1
								CAA3023827.1	

**Table 3:** Endogenous peptides identified in olive pomace by de novo sequencing. ALC, average local confidence; N, number of amino acids; Z, precursor charge state; *m*/*z*, precursor mass-to-charge ratio; RT, retention time; Area, peptide (relative) abundance shown as area under the curve of the detected peptide; ppm, precursor mass error; local confidence, local confidence score for each amino acid of the peptide identified by de novo sequencing. Oxidised methionine is indicated by a pair of parentheses enclosing the modification mass (+15.99). ND, not detected.

No.	Peptide	ALC (%)	N	Z	*m*/*z*	RT	Area	ppm	Local Confidence (%)
1	LFYHHH	90	6	2	427.2079	30.18	1.96 × 10^6^	−2.2	88 85 88 95 88 96
2	GDHSGPPR	89	8	2	411.6919	31.88	5.36 × 10^6^	−10.6	56 92 97 97 94 97 95 90
3	YCFHSAA	89	7	2	399.6636	32.80	4.63 × 10^5^	−5.0	96 95 91 88 84 85 88
4	YTM(+15.99)M(+15.99)LF	89	6	2	419.1794	23.63	4.06 × 10^6^	−0.8	92 90 92 76 93 93
5	AEFENFVAFVDK	88	12	2	708.3472	48.76	1.88 × 10^6^	2.2	40 72 98 99 95 96 93 95 99 98 96 82
6	RCYHEE	87	6	2	418.6729	49.92	ND	3.6	84 93 90 85 85 86
7	YVM(+15.99)FFL	87	6	2	418.2032	29.26	1.31 × 10^6^	−8.0	98 94 92 87 71 80
8	FFTYAY	87	6	2	406.1843	36.79	2.90 × 10^5^	−5.9	85 85 90 90 85 86
9	YEM(+15.99)YPY	87	6	2	441.1741	25.78	3.42 × 10^7^	2.7	82 86 80 92 82 97
10	LSVSAFNC	86	8	2	420.7043	28.04	6.54 × 10^5^	11.2	93 94 94 90 92 93 69 69

**Table 4:** Predicted activity of water-soluble LMW peptides extracted from olive pomace identified by Peaks DB using the Biopep-UWM database of bioactive peptides. ACE, angiotensin-converting enzyme; DPP III, dipeptidyl peptidase-III; DPP IV, dipeptidyl peptidase-IV; A, ratio of number of sequences with predicted activity to number of amino acids; B, potential biological activity of the peptide (µM^−1^) theoretically calculated from previous bioactive referenced data. A and B values obtained from the BIOPEP-UWM database.

No.	Peptide Sequence	Predicted Activity	No. Seq. with Activity	A	B
1	NSNALACCSA	ACE inhibitor	1	0.1000	0.0003
		Antioxidative	1	0.1000	
		DPP III inhibitor	1	0.1000	
		DPP IV inhibitor	3	0.1000	
		Activating ubiquitin-mediated proteolysis	1	0.1000	0.0001
2	GGDNGMNH	ACE inhibitor	4	0.5	0.0001
		DPP IV inhibitor	5	0.6250	0.0018
3	RSCQHRY	ACE inhibitor	1	0.1429	0.0136
		Antioxidative	1	0.1429	9.72 × 10^−6^
		DPP IV inhibitor	2	0.2857	8.47 × 10^−5^
4	LPPDNSH	ACE inhibitor	3	0.4286	0.0161
		alpha-glucosidase inhibitor	1	0.1429	
		DPP IV inhibitor	4	0.5714	0.0018
5	SLIPCCSS	ACE inhibitor (IP)	1	0.125	0.001
		DPP IV inhibitor	3	0.375	0.0003
		Regulator of phosphoglycerate kinase activity	1	0.125	
		Glucose uptake stimulating peptide	1	0.125	
6	SGDPTM(+15.99)YE	ACE inhibitor	4	0.2500	0.0001
		DPP IV inhibitor	4	0.2500	
7	IDHGCAKH	ACE inhibitor	1	0.125	1.98 × 10^−5^
		DPP IV inhibitor	1	0.125	
		antioxidative	1	0.125	
8	IPATDSQG	ACE inhibitor	3	0.375	0.0019
		DPP IV inhibitor	6	0.75	0.0029
9	MCTAAEM(+15.99)K	ACE inhibitor	1	0.0625	0.0001
		hypotensive	1	0.0625	
		DPP IV inhibitor	3	0.1875	6.64 × 10^−6^
10	GGGPGGGLGGPS	ACE inhibitor	5	0.8333	0.0008
		DPP IV inhibitor	5	0.8333	4.91 × 10^−5^
		PAM inhibitor	1	0.0833	
		peptide regulating the stomach mucosal membrane activity	3	0.3333	
		Antithrombotic peptide	3	0.3333	
		Prolyl endopeptidase inhibitor (antiamnestic)	3	0.3333	
11	QANACHH	antioxidative	1	0.1429	3.11 × 10^−2^
		DPP IV inhibitor	3	0.4286	
12	NSCCVCMVR	ACE inhibitor	1	0.1111	0.0021
		DPP IV inhibitor	2	0.2222	0.0001

**Table 5:** Predicted activity of water-soluble LMW peptides extracted from olive pomace identified by de novo sequencing using the Biopep-UWM database of bioactive peptides. ACE, angiotensin converting enzyme; DPP III, dipeptidyl peptidase-III; DPP IV, dipeptidyl peptidase-IV; A, ratio of number of sequences with predicted activity to number of amino acids; B, potential biological activity of the peptide (µM^−1^) theoretically calculated from previous bioactive referenced data. A and B values obtained from the BIOPEP-UWM database.

No.	De Novo Peptide	Predicted Activity	No. Seq. with Activity	A	B
1	LFYHHH	ACE inhibitor	3	0.5	0.0396
		Antioxidative	3	0.667	
		DPP III inhibitor	2	0.1667	
		DPP IV inhibitor	1	0.5000	
2	GDHSGPPR	ACE inhibitor	7	0.875	0.0380
		Antioxidative	1	0.1250	
		DPP III inhibitor	1	0.1250	
		DPP IV inhibitor	1	0.375	
		antiamnestic	1	0.1250	
		antithrombotic	1	0.1250	
		regulating	1	0.1250	
		alpha-glucosidase inhibitor	1	0.1250	
3	YCFHSAA	ACE inhibitor	3	0.2857	0.0731
		hypotensive	1	0.2857	1.519 × 10^−5^
		DPP IV inhibitor	2	0.1429	
4	YTM(+15.99)M(+15.99)LF	ACE inhibitor	2	0.3333	0.0008
		Antioxidative	1	0.1667	0.0003
		DPP IV inhibitor	4	0.6667	0.0036
5	AEFENFVAFVDK	ACE inhibitor	4	0.3333	0.0046
		DPP IV inhibitor	5	0.0833	0.0005
		CaMPDE inhibitor	1	0.4167	
		hypolipidemic	2	0.0833	
		renin inhibitor	1	0.0833	
6	RCYHEE	ACE inhibitor	1	0.1667	0.0325
		DPP III inhibitor	1	0.1667	
		DPP IV inhibitor	1	0.3333	
		stimulating	1	0.1667	
7	YVM(+15.99)FFL	ACE inhibitor	5	0.8333	0.0113
		DPP III inhibitor	5	0.1667	
		DPP IV inhibitor	1	0.8333	0.0006
8	FFTYAY	ACE inhibitor	4	0.5000	0.0119
		Antioxidative	4	0.6667	
		DPP IV inhibitor	4	0.6667	0.0002
		renin inhibitor	2	0.333	
9	YEM(+15.99)YPY	ACE inhibitor	3	0.5000	0.0014
		Antioxidative	1	0.1667	
		DPP IV inhibitor	5	0.8333	0.0007
		anti inflammatory	1	0.1667	
		alpha-glucosidase inhibitor	2	0.3333	0.0001
10	LSVSAFNC	ACE inhibitor	1	0.1250	0.0007
		DPP IV inhibitor	4	0.5000	

## Data Availability

The data presented in this study are available on request from the corresponding author.

## Supplementary Materials

The supporting information can be downloaded at: https://www.mdpi.com/article/10.3390/ijms25073962/s1.
