# Supplementary material for: Olive Pomace Extract Contains Low Molecular Weight Peptides and Possesses ACE Inhibitory Activity
Source: Int J Mol Sci. 2024 Apr 2;25(7):3962. doi: 10.3390/ijms25073962 (PMC11011677; doi:10.3390/ijms25073962)
Supplement: Supplementary file 1 [file ijms-25-03962-s001.zip › Supplemental S1.pdf]

Supplemental data S1. Endogenous peptides identified in olive pomace using Peaks database (DB) algorithm, filtered by -10lgP score >25 (30 peptides). -10lgP score, statistical significance of the peptide-precursor spectrum match; N, number of amino acids; m/z, precursor mass-to-charge ratio; Mass, peptide mass in Da; RT, retention time; ppm, precursor mass error; Area, peptide abundance shown as area under the curve of the detected peptide; Accession, the accession number of the highest-scoring protein containing this peptide. Oxidised methionine is indicated by a pair of parentheses enclosing the modification mass (+15.99).

Peptide	-10lgP	Mass	N	ppm	m/z	RT	Accession
NSNALACCSA	34.52	9.523.743	10	7.1	4.771.920	51.20	OE9A062433P1
GGDNGMNH	31.98	8.002.872	8	-4.5	4.011.449	51.36	OE9A085147P3:OE9A085147P2
RSCQHRY	30.05	9.484.348	7	0.4	4.752.191	21.09	OE9A113191P1
ISCHYLF	29.46	8.814.105	7	7.4	4.417.104	33.17	
LSQPCCHSGK	29.26	10.584.637	10	-11.6	5.302.275	55.41	
LPPDNSH	29.26	7.783.610	7	2.7	3.901.841	52.42	OE9A032896P1
NMHANPSH	28.12	9.063.766	8	1.6	4.541.908	50.91	
RNDASAHCQ	27.61	10.004.145	9	-8.2	5.012.031	56.67	
SLIPCCSS	27.21	8.083.459	8	-6.6	8.093.359	54.15	OE9A059381P2:OE9A059381P1:OE9A059381P3
CPASCCAAIS	26.84	9.243.503	10	-12.8	4.631.709	43.89	
SGDPTM(+15.99)YE	26.79	9.143.328	8	-11.8	4.581.616	50.95	OE9A048079P1
GATCPGCGGH	26.72	8.583.113	10	-14.4	4.301.516	79.60	
RCPGSHGR	26.50	8.684.086	8	1.9	4.352.060	41.65	
RHCCDRH	26.12	9.253.759	7	-7.1	4.636.872	43.55	
PPPAGAAVCG	26.03	8.384.007	10	-3.7	4.201.999	43.61	
GSGAPGGGGGGGGIPGA	25.89	11.815.425	17	-0.1	5.917.713	70.61	
NSPQHYSN	25.79	9.453.940	8	5.8	4.737.001	30.71	
MAHSGFKG	25.55	8.333.854	8	-5.9	4.176.925	33.43	
RGNDTSHV	25.51	8.844.100	8	-3.3	4.432.043	31.96	
IDHGCAKH	25.50	8.794.021	8	-1.2	4.407.025	42.72	OE9A062433P1
IPATDSQG	25.47	7.873.712	8	4.6	3.946.899	36.41	OE9A083314P2:OE9A083314P3:OE9A083314P1
NSGLGDGY	25.46	7.813.242	8	2.4	3.916.656	35.22	
SNYGGPGPS	25.38	8.343.508	9	-11.9	4.181.716	43.33	
TKEICCCM(+15.99)AHGSG	25.36	13.545.138	13	-8.9	6.782.499	74.39	
SNQACDHPT	25.33	9.713.767	9	-0.4	4.866.896	37.06	
MCTAAEM(+15.99)K	25.21	8.993.551	8	9.9	4.506.838	48.32	OE9A053685P3
GGGPGGGLGGPS	25.13	8.684.039	12	3.6	4.352.055	45.44	OE9A068558P1
QANACHH	25.10	7.793.133	7	-2.5	3.906.582	33.30	OE9A008961P3
VGDEGMHR	25.02	8.993.920	8	-0.6	4.506.976	28.97	
NSCCVCMVR	25.00	10.133.915	9	-4.6	5.076.946	42.64	OE9A009309P1
**total 30 peptides**							
