# Supplementary material for: Olive Pomace Extract Contains Low Molecular Weight Peptides and Possesses ACE Inhibitory Activity
Source: Int J Mol Sci. 2024 Apr 2;25(7):3962. doi: 10.3390/ijms25073962 (PMC11011677; doi:10.3390/ijms25073962)
Supplement: Supplementary file 1 [file ijms-25-03962-s001.zip › Supplemental S2.pdf]

Supplemental data S2. Proteins identified as sources of endogenous peptides detected in pomace olive. Protein -10lgP score, sum of the -10lgP scores of the protein's supporting peptides; Peptides, number of high-confidence supporting peptides; Cover (%), percentage of the protein sequence that is covered by the supporting peptides; Area, protein relative abundance; Protein identification, definitions of best-matched proteins identified by BLAST search *(Olea europaea)*; E-value (score), number of expected hits of similar quality that could be found randomly; Protein description, access to protein report.

**Potein acession**	**-10lgP**	**Peptides**	**Cover (%)**	**Average Mass (Da)**	**Protein identification (BLAST)**	**Area**	E-value	**% identity**	**Protein description**
OE9A062433P1	47.27	2	4	111435	anthocyanidin 3-O-glucosyltransferase	3.32×10^7	0.0	100%	CAA2981081.1
OE9A008961P3	45.24	2	1	816842	DUF946 domain-containing DUF1162 domain-containing Chorein_N domain-containing	3.23×10^5	0.0	100%	CAA2957578.1
OE9A051261P1	45.01	1	1	263828	myc box-dependent-interacting 1 isoform X1	1.66×10^5	0.0	100%	CAA2990894.1
OE9A053685P3	43.05	1	1	270051	white-brown complex homolog 30	4.66×10^5	0.0	100%	CAA2969019.1
OE9A085147P3	40.15	1	1	218083	phospholipase D gamma 1-like	1.53×10^6	0.0	100%	CAA2951337.1
OE9A032896P1	38.50	1	2	97821	Hypothetical predicted protein	3.26×10^6		100%	CAA2978878.1
OE9A087070P3	37.75	1	2	101228	bZIP transcription factor 16-like	1.58×10^6			CAA3013064.1
OE9A048079P1	35.98	1	2	91628	tryptophan aminotransferase-related 2 isoform X1	2.68×10^5	0.0	100%	CAA2969551.1
OE9A059381P1	35.55	1	1	173684	casein kinase 1 HD16	6.69×10^6	0.0	100%	CAA2934848.1
OE9A030742P1	35.55	2	3	183927	ATP-dependent DNA helicase DDM1 isoform X1	1.46×10^5			CAA3021981.1
OE9A009309P1	34.37	1	3	73583	probable E3 ubiquitin- ligase LOG2	1.31×10^6	0.0	100%	CAA3023827.1
OE9A068558P1	33.70	1	1	200100	dihydrolipoyllysine-residue succinyltransferase component of 2-oxoglutarate dehydrogenase complex 1	3.10×10^6	0.0	100%	CAA3014561.1
OE9A083314P1	33.29	1	1	186425	auxin response factor 6 isoform X1	1.42×10^6		100%	CAA2976018.1
OE9A030338P1	32.94	2	3	162671	Hypothetical predicted protein	2.94×10^6		100%	CAA3033027.1
OE9A079929P2	32.04	1	1	248672	lysine-specific demethylase JMJ18-like	ND		100%	CAA2934425.1
OE9A057492P1	31.15	1	2	108336	uncharacterized protein LOC111390123	2.78×10^7		100%	XP_022870890.1
OE9A113191P1	30.05	1	2	93640	putative RING-H2 finger protein ATL21A	ND	0.0	100%	XP_022855964.1
OE9A038478P1	30.04	1	1	163083	low affinity sulfate transporter 3	8.74×10^4		100%	CAA3018940.1
